# Facility‐Level Factors Associating Antenatal Corticosteroid Administration Rates and Subsequent Term Birth Rates: A Nationwide Cross‐Sectional Observational Study Using the 2020–2022 Perinatal Registry Database in Japan

**DOI:** 10.1111/jog.70237

**Published:** 2026-03-12

**Authors:** Kazuya Fuma, Takafumi Ushida, Takahiro Imaizumi, Sho Tano, Seiko Matsuo, Satoru Katsuki, Kenji Imai, Hiroaki Kajiyama, Tomomi Kotani

**Affiliations:** ^1^ Department of Obstetrics and Gynecology Nagoya University Graduate School of Medicine Nagoya Japan; ^2^ Center for Maternal‐Neonatal Care Nagoya University Hospital Nagoya Japan; ^3^ Department of Clinical Research Education Nagoya University Graduate School of Medicine Nagoya Japan; ^4^ Department of Obstetrics and Gynecology Hamamatsu University School of Medicine Hamamatsu Japan

**Keywords:** delivery, glucocorticoids, neurodevelopmental disorders, obstetric, premature birth

## Abstract

**Aim:**

The antenatal corticosteroid (ACS) administration rate in Japan is low. To achieve both high coverage and low overtreatment of ACS, understanding of facility‐level factors is important. This study aimed to identify facility‐level factors associated with ACS coverage and overtreatment and simulate the potential consequences of increased ACS use.

**Methods:**

This observational study used data from the 2020 to 2022 Perinatal Registry Database maintained by the Japan Society of Obstetrics and Gynecology. Primary outcomes were: (1) ACS administration rate among preterm births before 34 weeks (ACS/34w rate) and (2) proportion of term births among ACS recipients (term/ACS proportion). Multivariable regression analyses examined associations with facility‐level factors including perinatal care level, location, delivery volume, and prevalence of maternal conditions. A simulation estimated the impact of increasing ACS/34w rate to 80% in facilities with lower baseline rates.

**Results:**

In the facility‐level analysis of 244 facilities (376 717 records), the mean ACS/34w rate was 63.4%, and term/ACS proportion was 12.0%. The proportion of threatened preterm labor (coefficient: 4.4 [95% confidence interval: 2.1–6.7]), deliveries < 34 weeks (3.0 [0.1–5.8]), and cesarean section rate (−2.4 [−4.5 to −0.2]) were significantly associated with ACS/34w rate. ACS/34w rate (2.0 [0.8–3.3]), annual delivery volume (2.1 [0.6–3.5]), and cesarean section rate (1.5 [0.2–2.7]) were positively associated with term/ACS proportion, while perinatal care level was inversely associated (−3.5 [−6.3 to −0.6]). Simulation estimated 2311 additional ACS recipients and 465 term births per year.

**Conclusions:**

Facility‐level factors influence ACS coverage and overtreatment. These findings may inform strategies for optimizing ACS use.

## 1 Introduction

Appropriate administration of antenatal corticosteroids (ACS) improves neonatal outcomes in preterm infants and is widely recommended [1]. Although many high‐income countries report ACS administration rates exceeding 80%–90%, the rate in Japan remains substantially lower (64%) [2, 3]. Improving ACS coverage is therefore a national priority.

However, achieving high ACS administration rates alone does not ensure “appropriate” use. International reports indicate that only 20%–40% of ACS recipients deliver within the optimal 7‐day window, whereas 40%–45% deliver at term, raising concerns regarding overtreatment [4]. To address this, the Society for Maternal‐Fetal Medicine (SMFM) has proposed two balancing metrics: (1) the rate of optimally timed ACS among births before 34 weeks (optimal‐ACS/34w rate) and (2) the proportion of term births among ACS recipients (term/ACS proportion) [4].

Japan presents a distinctive profile: while the national ACS administration rate among preterm births before 34 weeks (ACS/34w rate) is relatively modest (64%), Japan shows comparatively favorable timing‐related indicators—over half of ACS recipients deliver within 7 days, and only 14.4% deliver at term [3]. However, it remains uncertain whether these favorable outcomes can be sustained as ACS use becomes more widespread in Japan. To our knowledge, no previous studies have demonstrated an association between the ACS/34w rate and the term/ACS proportion.

Evaluation of this association requires facility‐level analysis, as these ACS‐related metrics are proportions that cannot be defined or evaluated at the individual case level. In addition, facility‐level analysis may help identify institutional characteristics associated with appropriate ACS‐related metrics.

Accordingly, we used Japan's nationwide Perinatal Registry Database to: (1) identify facility‐level factors associated with ACS‐related metrics and (2) simulate the potential consequences of increased ACS use, focusing on the trade‐off between benefit and possible overtreatment. These findings offer foundational insights for optimizing ACS administration practices in Japan.

## 2 Methods

### 2.1 Study Design

This study is a nationwide, registry‐based observational analysis of births in Japan, utilizing the Perinatal Registry Database from 2020 to 2022. This study was conducted in accordance with the Declaration of Helsinki and all other relevant guidelines and regulations and was approved by the Institutional Ethics Boards of Nagoya University Hospital (approval number: 2024‐0467; November 11, 2024) and JSOG (approval number: 169; August 27, 2024). The requirement for informed consent was waived by both Institutional Ethics Boards in accordance with the Ethical Guidelines for Medical and Health Research Involving Human Participants in Japan.

### 2.2 Perinatal Registry Database

The Perinatal Registry Database is a national database maintained by the Japan Society of Obstetrics and Gynecology (JSOG) since 2001, which collects clinical information on all deliveries after 22 weeks of gestation from participating obstetric facilities. According to publicly available information from JSOG, in 2022, the database contained 215 662 babies from 427 facilities, covering 27.9% of all births in Japan. Participation is skewed toward tertiary centers, resulting in an overrepresentation of high‐risk pregnancies. Consequently, our calculations indicate that this database includes 67.0% of preterm births before 37 weeks and 95.3% of those before 32 weeks in Japan. Each record in the database includes 12 major categories: basic information, delivery details, obstetric complications, procedures, neonatal characteristics, placental and fetal findings, obstetric history, underlying maternal conditions, infections, medication use, fetal treatments, and neonatal mortality—comprising 330 data fields. Data collection process was substantially updated in 2020; therefore, we included all available records from the most recent 3 years (2020–2022).

Exclusion criteria for this study were as follows: duplicate records; stillbirths or perinatal deaths with unknown timing; neonates other than first‐born in multiple pregnancies; and records with missing values in key obstetric variables, including parity, history of infertility treatment, number of fetuses, neonatal sex, maternal age at delivery, gestational age at delivery, birth weight, intrapartum blood loss, and maternal survival status. These variables were mandatory fields in the registry system, and missing values were therefore considered indicative of data entry errors. Additionally, facilities with < 10 deliveries before 34 weeks per year were excluded from the analysis because facilities with small case numbers yield unstable estimates owing to small‐number variation.

To enhance data reliability, we excluded facility–year combinations with ACS administration rates > 2.5 median absolute deviations from the median as outliers (Figure 2) [5].

### 2.3 ACS Administration in Japan

The Japanese guideline recommends administering ACS (betamethasone, 12 mg intramuscularly, given twice 24 h apart, for a total dose of 24 mg) to accelerate fetal lung maturation and prevent intracranial hemorrhage in preterm infants [6]. This recommendation applies under the following conditions: when preterm birth between 24^+0^ and 34^+6^ weeks of gestation is expected within 1 week (recommendation level B) or when preterm birth between 22^+0^ and 23^+6^ weeks of gestation is expected within 1 week (recommendation level C). In Japan, betamethasone up to 24 mg is approved per pregnancy. Any additional doses are considered off‐label and are not covered by insurance. Furthermore, only betamethasone is approved for fetal maturation in preterm pregnancies; dexamethasone is off‐label for this indication.

According to a 2022 survey conducted by JSOG, 48% of pregnant women in Japan deliver at primary obstetric care facilities. When preterm birth is anticipated at such facilities, maternal transfer to a higher‐level facility is arranged, where ACS is administered.

### 2.4 Facility‐Level Factors

We evaluated the following facility‐level factors: perinatal care level assigned by the respective prefectural governments in Japan as of December 2022, categorized as Comprehensive Perinatal Care Centers, Regional Perinatal Care Centers, or nondesignated facilities. These designations are based on multiple criteria, including the availability of maternal–fetal intensive care units and neonatal intensive care units, emergency transport systems, such as doctor cars, sufficient blood product reserves, and many obstetricians on site 24 h a day. Comprehensive Perinatal Care Centers function as tertiary centers that manage extremely preterm births and highly complicated cases referred from other facilities. Other evaluated variables included location in a government‐designated city; annual delivery volume; annual average number of deliveries before 34 weeks; annual average proportion of maternal transfers received among preterm births before 34 weeks; cesarean section rates among preterm births before 34 weeks; and morbidity rates among preterm births before 34 weeks, including threatened preterm labor (TPL), hypertensive disorders of pregnancy (HDP), preterm premature rupture of membranes (PROM), placenta previa, multiple pregnancies, fetal growth restriction (FGR), and placental abruption. We used these rates specifically among births before 34 weeks because they better reflect facility‐level practices in the management of high‐risk pregnancies.

### 2.5 Outcomes

As ACS‐related metrics, we defined the following primary outcomes: (Outcome 1) the ACS/34w rate and (Outcome 2) the term/ACS proportion. Additionally, we assessed two secondary outcomes: (Outcome 3) the optimal‐ACS/34w rate (defined as administration within 7 days before delivery), and (Outcome 4) ACS administration rates among all deliveries.

Cases with documented ACS dose or ACS‐to‐delivery interval were treated as having received ACS, even if the registry checkbox indicating ACS administration was not filled. Because the total planned ACS dose is often uncertain at the time of the first administration, we did not analyze cases with a single dose or 12 mg of ACS as a separate group; instead, all such cases were included as ACS recipients. Regarding the definition of “optimal” timing, the SMFM has proposed a definition as administration within 6–168 h before delivery [4]. However, due to the current registry structure, we applied a 7‐day window as the definition of “optimal” timing in this analysis. In the JSOG Perinatal Registry Database, the interval between ACS administration and delivery is recorded as categorical data: within 48 h, 48 h to 7 days, 7 days to 1 month, and > 1 month.

### 2.6 Statistical Analyses

We used R (version 4.2.3) and RStudio (2024.12.0 Build 467) for statistical analyses. Proportion‐based facility‐level factors were calculated as annual means using patient‐level data from the JSOG Perinatal Registry Database, which includes facility identifiers. In a multivariable analysis, a separate model was constructed for each outcome. All facility‐level factors described above were included in each of the four models. Additionally, the ACS/34w rate (Outcome 1) was included as an explanatory variable in other models (Outcomes 2–4) to assess its association with these outcomes. Continuous variables were standardized to allow for comparison of coefficient magnitudes across variables. We also assessed multicollinearity in each model and confirmed that all variance inflation factors (VIFs) were below 3.

### 2.7 Sensitivity Analyses

In some extremely severe cases, clinicians may have no opportunity to administer ACS after admission. Therefore, as a sensitivity analysis, we repeated the analyses after excluding pregnancies complicated by placental abruption and those undergoing Grade 1 cesarean section.

### 2.8 Simulation Analysis

See Supporting Information Method.

## 3 Results

**FIGURE 1:**
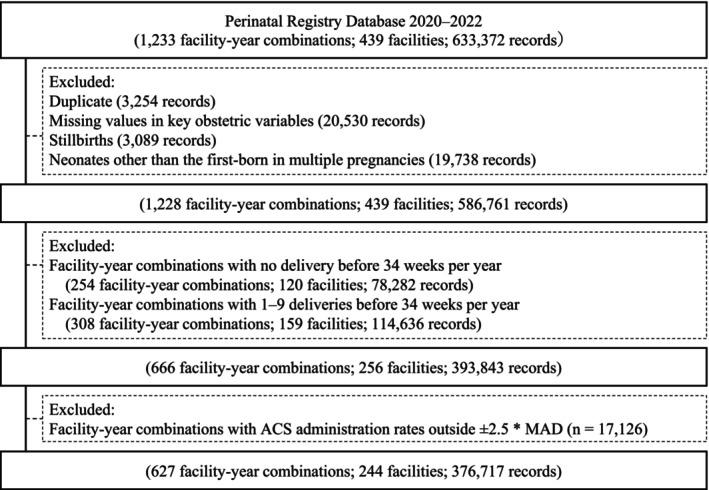
Flowchart of study facilities. ACS, antenatal corticosteroids; MAD, median absolute deviation.

**FIGURE 2:**
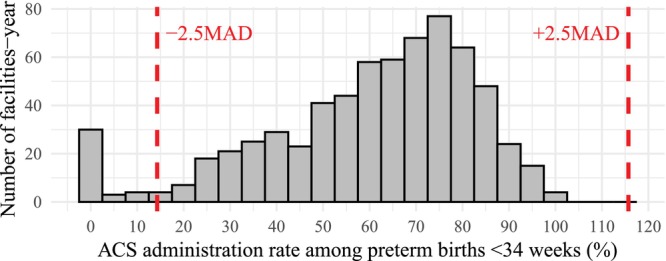
Histogram of the ACS/34w rate. Each facility–year counted as one observation. Dashed lines indicate ±2.5 times the median absolute deviation. ACS, antenatal corticosteroids; MAD, median absolute deviation.

Among the 439 facilities and 633 372 records included in the JSOG Perinatal Registry Database between 2020 and 2022, 244 facilities and 376 717 records met the criteria and were analyzed in this study (Figures 1 and 2). Table 1 presents the facility‐level factors. The majority of records (98.8%) originated from comprehensive or regional perinatal care centers, which typically manage high‐risk pregnancies in Japan. Among preterm births before 34 weeks, TPL was the most common complication [48.8% (standard deviation: ±16.2)], followed by preterm PROM, HDP, and FGR. Table 2 summarizes the ACS‐related metrics. Mean ACS/34w and optimal‐ACS/34w rates were 63.4% (±16.3) and 46.9% (±14.5), respectively. These rates remained consistent across different gestational ages within the preterm period. Among women who received ACS, the mean proportion of deliveries within 7 days was 56.4% (±16.9), and mean term/ACS proportion was 12.0% (±9.3).

**TABLE 1:** Summary of 244 facilities.

Category	Facility‐level factors	Mean (±SD), *n* (%)
Facility level	Comprehensive Perinatal Care Centers	104 (42.6%)
	Regional Perinatal Care Centers	137 (56.1%)
	Nondesignated facilities	3 (1.2%)
City type	Located in government‐designated city	90 (36.9%)
	Other locations	154 (63.1%)
Delivery volume by gestational age	Annual total deliveries	583.2 (±335.3)
	Full‐term deliveries	495.7 (±314.4)
	Deliveries < 37 weeks	87.6 (±41.1)
	Deliveries < 34 weeks	31.7 (±17.6)
	Deliveries < 32 weeks	18.3 (±12.8)
	Deliveries < 28 weeks	7.0 (±7.2)
Prevalence by gestational age	Full‐term deliveries	83.1% (±7.2)
	Preterm deliveries < 37w	16.9% (±7.2)
	Preterm deliveries < 34w	6.3% (±3.8)
	Preterm deliveries < 32w^a^	3.6% (±2.9)
	Preterm deliveries < 28w^b^	1.4% (±1.6)
Complications among preterm births < 34w	TPL	48.8% (±16.2)
	HDP	19.2% (±8.1)
	Preterm PROM	29.3% (±11.2)
	Placenta previa	4.8% (±3.6)
	Multiple pregnancy	11.4% (±5.5)
	FGR	14.3% (±7.1)
	Placental abruption	5.9% (±7.1)
Transfer and CS rate among preterm births < 34w	Maternal transfer	49.8% (±17.5)
	CS	73.0% (±11.3)

Abbreviations: CS, cesarean section; FGR, fetal growth restriction; HDP, hypertensive disorders of pregnancy; PROM, premature rupture of membranes; TPL, threatened preterm labor.

^a^ Three facilities were excluded from the calculation because no preterm deliveries before 32 weeks were recorded.

^b^ Forty‐one facilities were excluded from the calculation because no preterm deliveries before 28 weeks were recorded.

**TABLE 2:** Proportions of appropriate administrations of ACS.

Category	ACS‐related metrics	Mean (±SD)
ACS administration rate	Among all deliveries	6.3% (±4.0)
	Among preterm deliveries < 34w	63.4% (±16.3)
	Among preterm deliveries < 32w^a^	63.8% (±20.1)
	Among preterm deliveries < 28w^b^	60.0% (±27.3)
Optimally timed ACS administration rates	Among preterm deliveries < 34w	46.9% (±14.5)
	Among preterm deliveries < 32w^a^	46.8% (±17.4)
	Among preterm deliveries < 28w^b^	43.4% (±23.4)
Duration between ACS administration and delivery among ACS recipients	Within 48 h	30.2% (±15.5)
	48 h to 7 days	26.2% (±10.3)
	7 days to 1 month	21.9% (±10.2)
	Over 1 month	17.6% (±12.2)
	Data missing	4.0% (±12.6)
Total amount of ACS dosage among ACS recipients	12 mg	17.7% (±10.4)
	24 mg	76.4% (±15.0)
	36 mg	0.2% (±0.8)
	48 mg	1.0% (±2.3)
	Data missing	4.6% (±11.1)
Term birth among ACS recipients	Annual number	4.5 (±5.6)
	Proportion	12.0% (±9.3)

^a^ Three facilities were excluded from the calculation because no preterm deliveries before 32 weeks were recorded.

^b^ Forty‐one facilities were excluded from the calculation because no preterm deliveries before 28 weeks were recorded. ACS, antenatal corticosteroids.

We also summarized facility‐level characteristics of excluded facility–year combinations due to a small number of preterm deliveries before 34 weeks of gestation (< 10 deliveries per year) (Table S1). Compared with included facilities (≥ 10 preterm deliveries before 34 weeks per year), excluded facilities were less likely to be perinatal care centers, had fewer annual deliveries, and showed lower rates of obstetric complications among preterm births before 34 weeks of gestation. In addition, both the ACS/34w rate and the optimal‐ACS/34w rate were markedly lower in excluded facilities; notably, 59 of 159 excluded facilities had an ACS/34w rate of 0% (Table S2).

### 3.1 Facility‐Level Factors Associated With ACS/34w Rate

**FIGURE 3:**
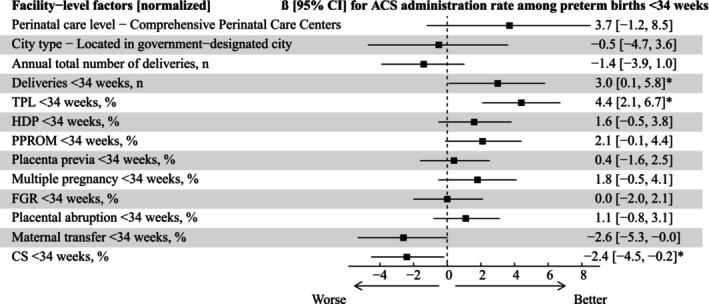
Relationships between facility‐level factors and the ACS/34w rate. All facility‐level factors shown in this figure were included as explanatory variables in multiple regression analyses after normalization. Perinatal care level was grouped as “Comprehensive Perinatal Care Centers” vs. “Other” due to the small number of nondesignated facilities. *Statistical significance was defined as a 95% confidence interval that did not cross zero. ACS, antenatal corticosteroids; CI, confidence interval; CS, cesarean section; FGR, fetal growth restriction; HDP, hypertensive disorders of pregnancy; PROM, premature rupture of membranes; TPL, threatened preterm labor.

Multivariable regression analysis revealed that several facility‐level factors were significantly associated with the ACS/34w rate (Figure 3). A one standard deviation (16.2 percentage points) increase in the prevalence of TPL among preterm births before 34 weeks was associated with a 4.4 (95% confidence interval [2.1–6.7]) percentage point increase in the ACS/34w rate. A one standard deviation (17.6) increase in the number of annual deliveries before 34 weeks was associated with a 3.0 (0.1–5.8) percentage point increase in the ACS/34w rate. In contrast, a one standard deviation (11.3 percentage points) increase in the cesarean section rate among preterm births before 34 weeks was associated with −2.4 (−4.5 to −0.2) percentage point changes in the ACS/34w rate. Although the perinatal care level of the facility was not significantly associated with the ACS/34w rate, it had one of the larger point estimates among the variables examined (3.7 [−1.2, 8.5] percentage points increase).

The sensitivity analysis excluding patients with placental abruption and those undergoing Grade 1 cesarean section also demonstrated similar trends; however, only one factor—the prevalence of TPL among preterm births before 34 weeks—remained statistically significant (Figure S1).

### 3.2 Facility‐Level Factors Associated With Term/ACS Proportion

**FIGURE 4:**
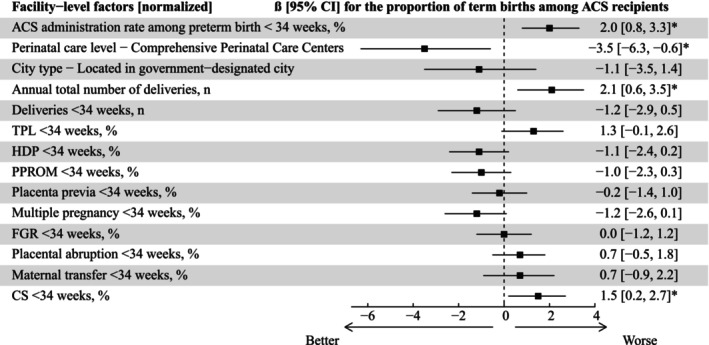
Relationships between ACS/34w rate and the term/ACS proportion. All facility‐level factors shown in this figure were included as explanatory variables in multiple regression analyses after normalization. Perinatal care level was grouped as “Comprehensive Perinatal Care Centers” vs. “Other” due to the small number of nondesignated facilities. *Statistical significance was defined as a 95% confidence interval that did not cross zero. ACS, antenatal corticosteroids; CI, confidence interval; CS, cesarean section; FGR, fetal growth restriction; HDP, hypertensive disorders of pregnancy; PROM, premature rupture of membranes; TPL, threatened preterm labor.

The ACS/34w rate was significantly positively associated with the term/ACS proportion (Figure 4). A one standard deviation (16.3 percentage points) increase in the ACS/34w rate was associated with a 2.0 [0.8–3.3] percentage points increase in the term/ACS proportion. Similarly, one standard deviation increase in the annual total number of deliveries (335.3 points) and cesarean section rate among preterm births before 34 weeks (11.3 percentage points) were associated with 2.1 [0.6–3.5] and 1.5 [0.2–2.7] percentage points increase in the term/ACS proportion, respectively. In contrast, designation as a Comprehensive Perinatal Care Center (highest level center in Japan) was the only significant variable negatively associated with this outcome, −3.5 [−6.3 to −0.6] percentage point change in the term/ACS proportion, and the magnitude of its estimated effect was greater than that of any other variable.

The results of the sensitivity analysis were largely unchanged (Figure S2).

### 3.3 Facility‐Level Factors Associated With Optimal‐ACS/34w Rate

The ACS/34w rate was significantly positively associated with the optimal‐ACS/34w rate (11.5 [10.2–12.8]) (Figure S3). Additionally, the annual number of preterm deliveries before 34 weeks was significantly negatively associated with this outcome, although the effect size was relatively small (−2.4 [−4.2 to −0.6]).

The results of the sensitivity analysis were largely unchanged; however, the prevalence of TPL among preterm births before 34 weeks newly showed a significant negative association with facility‐level optimal‐ACS/34w rates (−1.6 [−3.1 to −0.1]) (Figure S4).

### 3.4 Facility‐Level Factors Associated With ACS Administration Rates Among All Deliveries

The ACS/34w rate was significantly positively associated with the ACS administration rates among all deliveries (1.6 [1.2–1.9], Figure S5). Additionally, both the annual total number of deliveries (−1.8 [−2.2 to −1.5]) and number of deliveries before 34 weeks (2.2 [1.8–2.6]) were significantly associated with this outcome. Given that the three variables are mathematically interrelated, these associations are expected. The prevalence of placental abruption (0.4 [0.2–0.7]) and maternal transfer (0.6 [0.2–1.0]) was also significantly associated with this outcome; however, their estimated effects were small.

Although the overall findings remained similar in the sensitivity analysis, the association for the prevalence of FGR among preterm births before 34 weeks lost statistical significance (Figure S6).

### 3.5 Simulation of Increased ACS/34w Rate

Using the coefficients with nonstandardized variables (Table S3), we performed scenario‐based simulations to estimate the potential effects of increasing ACS/34w rate (Table 3). First, we assumed a typical obstetric facility in Japan with approximately 500 annual deliveries and 30 preterm births before 34 weeks. Under this scenario, if ACS coverage increases from 60% to 80%, an additional six eligible women with preterm births before 34 weeks would receive ACS treatment (Scenario 1). The number of optimally timed ACS before 34 weeks would increase by 4.2, overall ACS recipients would increase by 9.6, and term births among ACS recipients would increase by 2.4. Furthermore, if ACS/34w rate increases from 40% to 80%, an additional 12 eligible women with preterm births before 34 weeks would receive ACS treatment, including 8.6 more receiving optimally timed ACS and 4 more delivering at term following ACS exposure (Scenario 2).

**TABLE 3:** Simulated scenario and ACS‐related metrics.

	Scenario 1 facility with 500 annual deliveries and 30 preterm births < 34 weeks	Scenario 2 facility with 500 annual deliveries and 30 preterm births < 34 weeks	Scenario 3 All facilities with < 80% of the ACS/34w rate (123 234 deliveries/6577 preterm before 34 weeks)
Simulated increase in ACS administration rate before 34 weeks	60% → 80%	40% → 80%	< 80% → 80%
Estimated additional number in simulation			
Who administrated ACS and delivered before 34 weeks	+6.0 (18 → 24)	+12 (12 → 24)	+1211
Who administrated optimally timed ACS and delivered before 34 weeks	+4.2 (14 → 18.2)	+8.6 (9 → 17.6)	+851
Who administrated ACS (overall)	+9.6 (40 → 49.6)	+19.6 (20 → 39.6)	+2311
Who administrated ACS and delivered at term	+2.4 (5 → 7.4)	+4 (2 → 6.0)	+465

*Note:* Parentheses show baseline and postsimulation values, respectively.

Abbreviation: ACS, antenatal corticosteroids.

Next, we conducted a nationwide simulation in which all facilities included in this study with an ACS/34w rate < 80% were hypothetically set to an 80% administration rate (Scenario 3; 123 234 deliveries and 6577 preterm births before 34 weeks). Under this scenario, we estimated an increase of 2311 ACS recipients, including 1211 women delivering before 34 weeks, 851 with optimally timed ACS, and 465 delivering at term (Table 3). Based on effect estimates from Cochrane reviews and a large observational study [7, 8], these additional ACS administrations would be expected to prevent approximately 53 perinatal deaths, 32 cases of IVH, and 88 cases of childhood developmental delay per year. Conversely, the associated increase in term births following ACS exposure would correspond to an estimated 12 additional cases of any mental and behavioral disorder per year.

## 4 Discussion

The main findings of this study are as follows: several facility‐level factors were independently associated with key ACS‐related metrics, the ACS/34w rate, and term/ACS proportion. Notably, a higher ACS/34w rate was significantly associated with a higher term/ACS proportion. A simulation scenario in which the ACS/34w rate was increased suggested that the number of patients expected to benefit from ACS would exceed the number potentially exposed to associated risks.

In this study, the mean ACS/34w rate was 63.4%, which is consistent with previous reports based on another Japanese registry database managed by the Neonatal Research Network of Japan [2]. In contrast, Shigemi et al. reported a lower rate of 46.2% using a national insurance claims database [9]. However, their study included only patients treated with ritodrine and excluded all those treated with magnesium sulfate, which likely led to differences in patient characteristics. In our multivariable analysis, the prevalence of TPL among preterm births before 34 weeks was positively associated with the mean ACS/34w rate and had the largest coefficient among the standardized variables, suggesting it was the most influential factor. This association remained robust in our sensitivity analyses. Therefore, ensuring appropriate ACS administration for patients with TPL may be key to improving ACS use. As a next step, it will be important to investigate how facilities with high ACS/34w rates screen for and manage TPL in order to identify effective practices. Furthermore, facilities excluded because of small annual numbers (1–9) of preterm deliveries had lower ACS administration rates before 34 weeks, with approximately one‐third showing a rate of 0%. However, these facilities are unlikely to function as referral centers for deliveries before 34 weeks of gestation, and such deliveries in these settings may represent sporadic or nonsystematic events rather than routine management of high‐risk pregnancies. This potential case–mix heterogeneity warrants cautious interpretation of the observed ACS administration rates in the excluded facilities.

The mean rate of optimal‐ACS/34w rate was 46.9%. Shigemi et al. reported a lower rate of this metric (23.9%) in Japan [9]; however, as noted above, their inclusion criteria were limited by medication‐based case selection. To our knowledge, this study is the first to report a nationwide estimate of optimal‐ACS/34w rate in Japan without restricting the study population based on clinical presentation. Although previous studies from other countries have reported optimal‐ACS/34w rates of 23% to 40.7%—varying by gestational age and the definition of “optimal” timing—our findings indicate that Japan achieves comparable timing quality, despite lower overall usage [10, 11, 12].

The cesarean section rate among preterm births before 34 weeks was negatively associated with the ACS/34w rate and positively associated with the term/ACS proportion. This pattern indicates suboptimal ACS use, and the cesarean section rate was the only variable showing this pattern. The cesarean section rate may reflect both the severity of the cases managed and institutional staffing capacity. Previous studies have reported that facilities with limited staff availability tend to have higher cesarean section rates [13, 14]. In such settings, it may be challenging to monitor high‐risk patients long enough to complete the full course of ACS. However, as fetal benefits of ACS may begin as early as 3 h after administration, initiating ACS treatment should still be considered, even when completing the full course is not feasible [12]. Incorporating this information into clinical guidelines may help improve ACS administration practices. Further studies are warranted to clarify the factors underlying the association between cesarean section rates and ACS‐related metrics.

In this study, the term/ACS proportion was 12.0%, which is substantially lower than the rates of 32%–45% reported in other countries [15, 16, 17, 18]. The most influential factor associated with this outcome was perinatal care level. Facilities with the highest perinatal care level had a 3.5 percentage point lower term/ACS proportion compared with the other facilities. Although not statistically significant, these facilities were also associated with a 3.7 percentage point higher ACS/34w rate. ACS implementation practices at higher‐level facilities may provide valuable insights for improving the appropriate use of ACS. Future survey‐based studies are warranted to better understand institutional policies and decision‐making processes.

In our simulated scenario of enhanced ACS administration, although the number of term births among ACS recipients may increase, the estimated reductions in perinatal mortality, IVH, and developmental delay in childhood are considered to be greater in magnitude than the potential increase in any mental and behavioral disorders. Furthermore, setting a target ACS/34w rate of 80% appears feasible, as many facilities already report rates close to this threshold (Figure 2). In contrast, only five facilities in the JSOG Perinatal Registry Database exceeded a 90% ACS administration rate—a benchmark achieved by some high‐income countries. Given the small number of such facilities in Japan, we did not simulate a 90% target scenario, as doing so would raise concerns about the stability and generalizability of the estimates. Moreover, as indicated by the quadratic term for *ΔR* in our simulation formula, the number of term births following ACS administration is expected to increase at an accelerating rate as the target ACS rate rises. This suggests that extremely high ACS coverage could lead to a steep increase in term births after ACS exposure. To ensure that ACS is administered based on an appropriate balance between risks and benefits, potential disadvantages—including the possibility of neurodevelopmental impairment among term‐born infants—should continue to be investigated.

Although the reasons for this international discrepancy are not fully elucidated by the present study, differences in the management of TPL may be a contributing factor. In many countries, short‐term tocolysis is common, with hospitalization typically lasting only 1–3 days [19, 20]. In such settings, ACS is often initiated concurrently with maternal transfer, resulting in higher administration rates and a greater likelihood of overtreatment. In Japan, however, long‐term tocolysis is more common, allowing clinicians to observe the clinical course over several days and determine ACS indications accordingly [21]. This practice may reduce overtreatment, but may increase the risk of missed opportunities. Thus, Japan's unique management approach to TPL may partly explain the observed characteristics of ACS‐related metrics in this study. A Cochrane network meta‐analysis has shown that many tocolytic drugs are probably or possibly effective in delaying preterm birth for 7 days, and the World Health Organization's position has shifted from not recommending tocolysis in 2015 to accepting acute and maintenance therapy with nifedipine in 2022 [22, 23]. The present study speculates the need for further studies on the relationship between tocolysis strategies and appropriate timing of ACS administration.

Several projects have successfully increased the administration rate of ACS or magnesium sulfate for neuroprotection [24, 25, 26]. Common components of these projects included awareness campaigns, provider training, knowledge updates, and audit and feedback processes. Whether such multifaceted strategies can also help prevent overtreatment remains to be determined. Insights into facility‐level factors associated with the term/ACS proportion, as highlighted in this study, may inform more targeted and balanced approaches to improving ACS administration practices.

A major strength of this study lies in its use of a nationwide registry database and the application of multivariable regression analysis at the facility level. Our study incorporated a broader set of facility‐level covariates, including delivery volume and disease prevalence, and identified a significant positive association between the ACS/34w rate and term/ACS proportion.

A limitation of this study is the potential underreporting of ACS administration due to variability in data entry practices across facilities. In the JSOG Perinatal Registry Database, ACS administration status is not a mandatory field, and some facilities may have failed to consistently document this information. To minimize the impact of such reporting bias, we excluded facilities with anomalously low ACS administration rates, as visualized in Figure 2. This approach helped ensure a more reliable estimation of ACS use nationwide. Another limitation is lack of data on the reasons for administering or not administering ACS. We hope updates to the registry will include such information to facilitate more detailed analyses and support improvements in appropriate ACS use. In this study, we conducted sensitivity analyses excluding pregnancies complicated by placental abruption and those undergoing Grade 1 cesarean section, and the results for most facility‐level factors remained robust. These findings suggest that interfacility differences in ACS administration rates may be influenced more by facility‐level factors than by the presence of extremely severe cases in which clinicians have no opportunity to administer ACS. Finally, although our estimates of the risks and benefits associated with increased ACS use were based on absolute differences reported in the Cochrane review and the observational study, these values may not fully reflect the characteristics of the Japanese population. Nonetheless, they provide a useful reference point for considering the overall impact of improving ACS coverage.

In conclusion, using Japan's nationwide Perinatal Registry Database, we found that the ACS/34w rate was significantly associated with the term/ACS proportion. Facility‐level factors, such as perinatal care level, prevalence of TPL, and cesarean section rate, could be associated with both ACS‐related metrics. Understanding how facilities with a high prevalence of TPL or those with advanced perinatal care levels manage ACS administration may provide insights into strategies for improving ACS use in Japan. Our simulation based on multivariable regression analysis speculated that increasing the ACS/34w rate to 80% may be justified from a risk–benefit perspective. These findings may contribute to the development of targeted implementation strategies to promote appropriate and effective ACS use. Further studies are warranted to identify effective strategies for improving ACS administration practices.

## Author Contributions

**Kazuya Fuma:** conceptualization, software, methodology, formal analysis, investigation, writing – original draft, funding acquisition. **Takafumi Ushida:** conceptualization, investigation, project administration, writing – review and editing. **Takahiro Imaizumi:** data curation, formal analysis, investigation, writing – review and editing. **Sho Tano:** investigation, data curation, writing – review and editing. **Seiko Matsuo:** investigation, data curation, writing – review and editing. **Satoru Katsuki:** writing – review and editing. **Kenji Imai:** writing – review and editing. **Hiroaki Kajiyama:** writing – review and editing, supervision. **Tomomi Kotani:** writing – review and editing, supervision.

## Funding

This work was supported by the Japan Society for the Promotion of Science (24K23509).

## Ethics Statement

This study was conducted in accordance with the Declaration of Helsinki and all other relevant guidelines and regulations and was approved by the Institutional Ethics Boards of Nagoya University Hospital (approval number: 2024‐0467; November 11, 2024) and JSOG (approval number: 169; August 27, 2024).

## Consent

The requirement for informed consent was waived by both Institutional Ethics Boards in accordance with the Ethical Guidelines for Medical and Health Research Involving Human Participants in Japan. No written consent has been obtained from the patients as there is no patient identifiable data included.

## Conflicts of Interest

Dr. Hiroaki Kajiyama is the Editor‐in‐Chief of the journal and the co‐author of this article. They were excluded from the peer‐review process and all editorial decisions related to the acceptance and publication of this article. Peer review was handled independently by the JOG Journal editorial office to minimize bias. The authors declare no conflicts of interest.

## Supporting information


**File S1:** Raw facility‐level data included in the analysis.

**Figure S1:** Association between facility‐level factors and the ACS/34w rate (sensitivity analysis excluding patients with placental abruption and those undergoing grade 1 cesarean section).

**Figure S2:** Association between ACS/34w rate and the term/ACS proportion (sensitivity analysis excluding patients with placental abruption and those undergoing grade 1 cesarean section).

**Figure S3:** Association between ACS/34w rate and optimal‐ACS/34w rate.

**Figure S4:** Association between ACS/34w rate and optimal‐ACS/34w rate (sensitivity analysis excluding patients with placental abruption and those undergoing grade 1 cesarean section).

**Figure S5:** Association between ACS/34w rate and ACS administration rates among all deliveries.

**Figure S6:** Association between ACS/34w rate and ACS administration rates among all deliveries (sensitivity analysis excluding patients with placental abruption and those undergoing grade 1 cesarean section).

**Table S1:** Characteristics of facilities which match our exclusion criteria “Facility–year combinations with 1–9 deliveries before 34 weeks per year.”

**Table S2:** ACS‐related metrics of facilities which match our exclusion criteria “Facility–year combinations with 1–9 deliveries before 34 weeks per year.”

**Table S3:** Relationships between ACS‐related indicators and facility‐level factors (nonnormalized).

**Data S1:** jog70237‐sup‐0011‐Supinfo.docx.

## Data Availability

The raw data of the facilities included in this study are provided in File S1.
