# Supplementary material for: Facility‐Level Factors Associating Antenatal Corticosteroid Administration Rates and Subsequent Term Birth Rates: A Nationwide Cross‐Sectional Observational Study Using the 2020–2022 Perinatal Registry Database in Japan
Source: J Obstet Gynaecol Res. 2026 Mar 12;52(3):e70237. doi: 10.1111/jog.70237 (PMC12982006; doi:10.1111/jog.70237)
Supplement: Supplementary file 3 — Figure S2: Association between ACS/34w rate and the term/ACS proportion (sensitivity analysis excluding patients with placental abruption and those undergoing grade 1 cesarean section). [file JOG-52-0-s010.docx]

**Supporting Information Figure S2.**

Association between ACS/34w rate and the term/ACS proportion (Sensitivity analysis excluding patients with placental abruption and those undergoing grade A cesarean section)


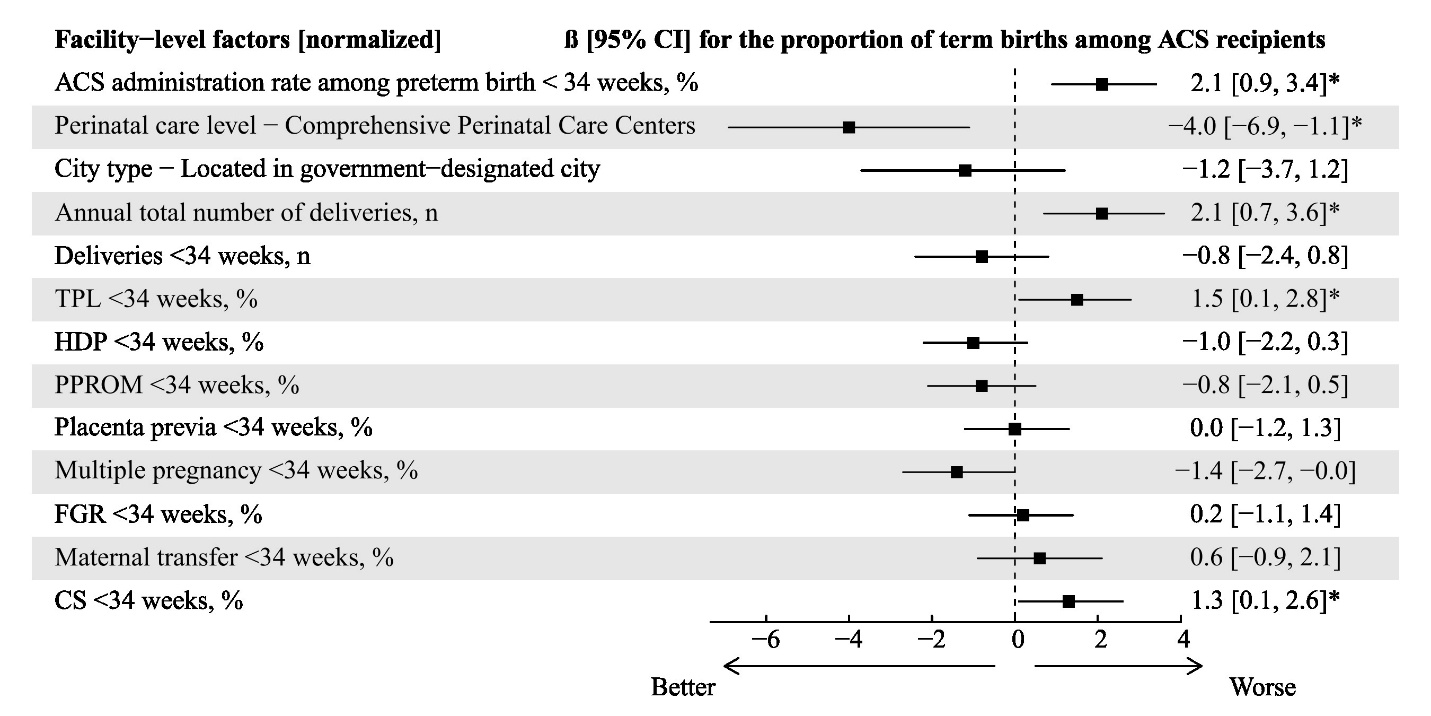


All facility-level factors shown in this figure were included as explanatory variables in multiple regression analyses after normalization. Perinatal care level was grouped as “Comprehensive Perinatal Care Centers” vs. “Other” due to the small number of non-designated facilities. *Statistical significance was defined as a 95% confidence interval that did not cross zero.

CI, confidence interval; ACS, antenatal corticosteroids; TPL, threatened preterm labor; HDP, hypertensive disorders of pregnancy; PPROM, preterm prelabor rupture of membranes; FGR, fetal growth restriction; CS, cesarean section
