# Supplementary material for: Facility‐Level Factors Associating Antenatal Corticosteroid Administration Rates and Subsequent Term Birth Rates: A Nationwide Cross‐Sectional Observational Study Using the 2020–2022 Perinatal Registry Database in Japan
Source: J Obstet Gynaecol Res. 2026 Mar 12;52(3):e70237. doi: 10.1111/jog.70237 (PMC12982006; doi:10.1111/jog.70237)
Supplement: Supplementary file 8 — Table S1: Characteristics of facilities which match our exclusion criteria “Facility–year combinations with 1–9 deliveries before 34 weeks per year.” [file JOG-52-0-s006.docx]

**Supporting Information Table S1.**

Characteristics of facilities which match our exclusion criteria “Facility-year combinations with 1–9 deliveries before 34 weeks per year”

|  | **Deliveries before 34 weeks per year per facility** | |  |
| --- | --- | --- | --- |
|  | **≥10** | **1–9** |  |
| Facility-year combinations; facilities; records, n | 666; 256; 393,843 | 308; 159; 114,636 | p |
| Facility level |  |  | <0.001 |
| Comprehensive Perinatal Care Centers, n (%) | 107 (41.8) | 8 (5.0) |  |
| Regional Perinatal Care Centers, n (%) | 146 (57.0) | 96 (60.4) |  |
| Non-designated Facilities, n (%) | 3 (1.2) | 55 (34.6) |  |
| City type |  |  | 0.991 |
| Located in government-designated city, n (%) | 90 (35.2) | 55 (34.6) |  |
| Other locations, n (%) | 166 (64.8) | 104 (65.4) |  |
| Delivery volume by gestational age |  |  |  |
| Annual total deliveries, n | 511.8 [370.4, 690.6] | 334.0 [236.0, 480.8] | <0.001 |
| Full-term deliveries, n | 433.2 [302.0, 591.3] | 302.0 [211.0, 451.9] | <0.001 |
| Deliveries <37 weeks, n | 75.8 [58.1, 107.2] | 27.5 [18.6, 39.0] | <0.001 |
| Deliveries <34 weeks, n | 26.5 [17.2, 40.7] | 3.7 [1.4, 7.0] | <0.001 |
| Deliveries <32 weeks, n | 14.7 [8.0, 25.7] | 1.0 [0.5, 2.2] | <0.001 |
| Deliveries <28 weeks, n | 4.7 [1.0, 10.0] | 0.0 [0.0, 0.3] | <0.001 |
| Prevalence by gestational age |  |  |  |
| Full-term deliveries, % | 84.0 [79.1, 88.2] | 92.5 [89.1, 95.1] | <0.001 |
| Preterm deliveries <37w, % | 16.0 [11.8, 20.9] | 7.5 [4.9, 10.9] | <0.001 |
| Preterm deliveries <34w, % | 5.4 [3.6, 7.7] | 1.1 [0.5, 1.8] | <0.001 |
| Preterm deliveries <32w,^a^ % | 2.9 [1.7, 4.7] | 0.3 [0.1, 0.7] | <0.001 |
| Preterm deliveries <28w,^a^ % | 0.9 [0.2, 1.9] | 0.0 [0.0, 0.1] | <0.001 |
| Complications among preterm births <34w |  |  |  |
| TPL, % | 48.4 [37.6, 58.7] | 40.0 [11.1, 60.3] | 0.001 |
| HDP, % | 19.1 [14.2, 23.2] | 6.7 [0.0, 23.6] | <0.001 |
| Preterm PROM, % | 29.1 [21.4, 35.5] | 12.5 [0.0, 31.1] | <0.001 |
| Placenta previa, % | 4.2 [2.4, 6.4] | 0.0 [0.0, 0.0] | <0.001 |
| Multiple pregnancy, % | 10.9 [7.5, 14.9] | 0.0 [0.0, 10.8] | <0.001 |
| FGR, % | 13.9 [8.9, 18.6] | 0.0 [0.0, 11.1] | <0.001 |
| Placental abruption, % | 4.9 [2.7, 7.6] | 0.0 [0.0, 10.7] | <0.001 |
| Transfer and cesarean section rate among preterm births <34w |  |  |  |
| Maternal transfer, % | 50.0 [38.1, 62.0] | 19.4 [0.0, 42.0] | <0.001 |
| CS, % | 73.4 [65.5, 80.0] | 61.7 [45.1, 77.6] | <0.001 |

In facilities with 1–9 preterm deliveries before 34 weeks of gestation per year, 59 of 159 facilities had an ACS administration rate of 0% among eligible preterm deliveries. Given this high frequency of zero values, the distribution was highly skewed, making it difficult to define statistical outliers using the ±2.5 × MAD criterion. Therefore, the values shown in this table were calculated prior to applying this final exclusion criterion (Figure 1). Given the non-normal distribution of several variables, continuous variables are summarized as median [IQR]. The Mann–Whitney U test was used for comparisons of continuous variables, and Fisher’s exact test was used for categorical variables.

^a^ Facility–year combinations with no deliveries at the corresponding gestational period were excluded from the analysis.

IQR, interquartile range; ACS, antenatal corticosteroids; TPL, threatened preterm labor; HDP, hypertensive disorders of pregnancy; PROM, premature rupture of membranes; FGR, fetal growth restriction; CS, cesarean section
