# Supplementary material for: Facility‐Level Factors Associating Antenatal Corticosteroid Administration Rates and Subsequent Term Birth Rates: A Nationwide Cross‐Sectional Observational Study Using the 2020–2022 Perinatal Registry Database in Japan
Source: J Obstet Gynaecol Res. 2026 Mar 12;52(3):e70237. doi: 10.1111/jog.70237 (PMC12982006; doi:10.1111/jog.70237)
Supplement: Supplementary file 9 — Table S2: ACS‐related metrics of facilities which match our exclusion criteria “Facility–year combinations with 1–9 deliveries before 34 weeks per year.” [file JOG-52-0-s005.docx]

**Supporting Information Table S2.**

ACS-related metrics of facilities which match our exclusion criteria “Facility-year combinations with 1–9 deliveries before 34 weeks per year”

|  | **Deliveries before 34 weeks per year per facility** | |  |
| --- | --- | --- | --- |
|  | **≥10** | **1–9** |  |
| Facility-year combinations; facilities; records, n | 666; 256; 393,843 | 308; 159; 114,636 | p |
| ACS administration rate |  |  |  |
| Among all births, % | 5.5 [2.9, 8.1] | 0.8 [0.1, 2.2] | <0.001 |
| Among preterm births <34w, % | 64.5 [49.3, 74.9] | 28.6 [0.0, 54.0] | <0.001 |
| Among preterm births <32w^a^, % | 66.3 [48.9, 77.5] | 0.0 [0.0, 50.0] | <0.001 |
| Among preterm births <28w^a^, % | 65.6 [42.0, 77.4] | 0.0 [0.0, 58.3] | <0.001 |
| Optimally timed ACS administration rates |  |  |  |
| Among preterm births <34w, % | 46.6 [33.6, 57.3] | 22.2 [0.0, 49.0] | <0.001 |
| Among preterm births <32w^a^, % | 47.0 [32.7, 57.1] | 0.0 [0.0, 39.4] | <0.001 |
| Among preterm births <28w^a^, % | 44.4 [26.4, 57.7] | 0.0 [0.0, 33.3] | <0.001 |
| Duration between ACS administration and delivery among ACS recipients |  |  |  |
| Within 48 hours, % | 27.8 [18.7, 36.7] | 21.8 [10.8, 43.3] | 0.119 |
| 48 hours to 7 days, % | 25.4 [20.0, 31.7] | 16.7 [0.0, 33.3] | <0.001 |
| 7 days to 1 month, % | 21.8 [15.8, 28.2] | 19.7 [7.0, 33.3] | 0.133 |
| Over 1 month, % | 15.9 [8.6, 24.7] | 16.7 [0.0, 36.2] | 0.656 |
| Data missing, % | 0.0 [0.0, 0.0] | 0.0 [0.0, 0.0] | 0.198 |
| Total amount of ACS dosage among ACS recipients |  |  |  |
| 12mg, % | 16.3 [10.7, 22.1] | 11.7 [0.0, 25.3] | 0.010 |
| 24mg, % | 79.0 [71.8, 86.9] | 80.0 [66.7, 100.0] | 0.375 |
| 36mg, % | 0.0 [0.0, 0.0] | 0.0 [0.0, 0.0] | 0.003 |
| 48mg, % | 0.0 [0.0, 1.2] | 0.0 [0.0, 0.0] | <0.001 |
| Data missing, % | 0.0 [0.0, 3.5] | 0.0 [0.0, 2.8] | 0.019 |
| Term birth among ACS recipients |  |  |  |
| Annual number, n | 3.0 [1.0, 5.0] | 0.3 [0.0, 1.0] | <0.001 |
| Proportion, % | 10.6 [5.6, 16.7] | 14.3 [0.0, 33.4] | 0.185 |

In facilities with 1–9 preterm deliveries before 34 weeks of gestation per year, 59 of 159 facilities had an ACS administration rate of 0% among eligible preterm deliveries. Given this high frequency of zero values, the distribution was highly skewed, making it difficult to define statistical outliers using the ±2.5 × MAD criterion. Therefore, the values shown in this table were calculated prior to applying this final exclusion criterion (Figure 1). Given the non-normal distribution of several variables, continuous variables are summarized as median [IQR]. The Mann–Whitney U test was used for comparisons of continuous variables, and Fisher’s exact test was used for categorical variables.

^a^ Facility–year combinations with no deliveries at the corresponding gestational period were excluded from the analysis.

IQR, interquartile range; ACS, antenatal corticosteroids; TPL, threatened preterm labor; HDP, hypertensive disorders of pregnancy; PROM, premature rupture of membranes; FGR, fetal growth restriction; CS, cesarean section
