# Supplementary material for: Facility‐Level Factors Associating Antenatal Corticosteroid Administration Rates and Subsequent Term Birth Rates: A Nationwide Cross‐Sectional Observational Study Using the 2020–2022 Perinatal Registry Database in Japan
Source: J Obstet Gynaecol Res. 2026 Mar 12;52(3):e70237. doi: 10.1111/jog.70237 (PMC12982006; doi:10.1111/jog.70237)
Supplement: Supplementary file 10 — Table S3: Relationships between ACS‐related indicators and facility‐level factors (nonnormalized). [file JOG-52-0-s002.docx]

**Supporting Information Table S3.**

Relationships Between ACS-Related Indicators and Facility-Level Factors (non-normalized)

|  | β [95% CI] | | |
| --- | --- | --- | --- |
| Facility-level factors (non-normalized) | **Outcome 2**  The proportion of term births among ACS recipients, % | **Outcome 3**  Optimally timed ACS administration rates among preterm births before 34 weeks, % | **Outcome 4** ACS administration rates among all deliveries, % |
| ACS administration rate among preterm births < 34 weeks, % | 0.125 [0.049, 0.200]* | 0.703 [0.622, 0.783]* | 0.096 [0.076, 0.115]* |
| Perinatal care level - Comprehensive Perinatal Care Centers | −3.470 [−6.319, −0.621]* | 0.379 [−2.654, 3.411] | −0.105 [−0.833, 0.623] |
| City type - Located in government-designated city | −1.054 [−3.482, 1.374] | −2.153 [−4.738, 0.432] | 0.449 [−0.171, 1.070] |
| Annual total number of deliveries, n | 0.006 [0.002, 0.010]* | −0.002 [−0.006, 0.003] | −0.005 [−0.007, −0.004]* |
| Deliveries <34 weeks, n | −0.068 [−0.163, 0.028] | −0.138 [−0.240, −0.036]* | 0.125 [0.101, 0.150]* |
| TPL <34 weeks, % | 0.078 [−0.005, 0.162] | −0.082 [−0.171, 0.007] | 0.002 [−0.019, 0.024] |
| HDP <34 weeks, % | −0.135 [−0.291, 0.020] | 0.109 [−0.056, 0.275] | −0.021 [−0.061, 0.018] |
| PPROM <34 weeks, % | −0.089 [−0.206, 0.028] | 0.104 [−0.021, 0.228] | −0.025 [−0.055, 0.004] |
| Placenta previa <34 weeks, % | −0.066 [−0.394, 0.262] | −0.110 [−0.459, 0.240] | −0.021 [−0.105, 0.063] |
| Multiple pregnancy <34 weeks, % | −0.223 [−0.463, 0.018] | −0.105 [−0.361, 0.152] | −0.015 [−0.076, 0.047] |
| FGR <34 weeks, % | 0.004 [−0.164, 0.173] | −0.099 [−0.279, 0.080] | 0.008 [−0.035, 0.051] |
| Placental abruption <34 weeks, % | 0.096 [−0.065, 0.258] | 0.017 [−0.155, 0.189] | 0.063 [0.021, 0.104]* |
| Maternal transfer <34 weeks, % | 0.039 [−0.049, 0.126] | −0.023 [−0.117, 0.070] | 0.034 [0.012, 0.056]* |
| CS <34 weeks, % | 0.128 [0.019, 0.238]* | −0.030 [−0.147, 0.086] | 0.001 [−0.027, 0.030] |

All facility-level factors shown in this table were included as explanatory variables in multiple regression analyses without normalization. Perinatal care level was grouped as “Comprehensive Perinatal Care Centers” vs. “Other” due to the small number of non-designated facilities. *Statistical significance was defined as a 95% confidence interval that did not cross zero.

CI, confidence interval; ACS, antenatal corticosteroids; TPL, threatened preterm labor; HDP, hypertensive disorders of pregnancy; PPROM, preterm prelabor rupture of membranes; FGR, fetal growth restriction; CS, cesarean section
