# Supplementary material for: Facility‐Level Factors Associating Antenatal Corticosteroid Administration Rates and Subsequent Term Birth Rates: A Nationwide Cross‐Sectional Observational Study Using the 2020–2022 Perinatal Registry Database in Japan
Source: J Obstet Gynaecol Res. 2026 Mar 12;52(3):e70237. doi: 10.1111/jog.70237 (PMC12982006; doi:10.1111/jog.70237)
Supplement: Supplementary file 11 — Data S1: jog70237‐sup‐0011‐Supinfo.docx. [file JOG-52-0-s008.docx]

**Supporting Information Method**

**Simulation analysis**

For simulation purpose, we used non-standardized coefficients to enable interpretation on the original scale. Based on the regression coefficients of the ACS/34w rate in models for Outcomes 2–4, we performed two scenario-based simulations. First, we assumed a typical obstetric facility in Japan with approximately 500 annual deliveries and 30 preterm births before 34 weeks. Term birth rate among ASC recipients was set to 12%. Under this scenario, we estimated additional number of cases for three outcomes when the ACS administration rate increased from either 40% or 60% to 80%: Three outcome estimations and formulas were as below:

(1) Estimated additional number of optimally timed ACS administration among preterm births before 34 weeks

= Preterm births before 34 weeks × (*B_opt_* + *c_opt_* × *∆R*) − Preterm births before 34 weeks × *B_opt_*

(2) Estimated additional number of ACS administration among all deliveries

= Annual deliveries × (*B_all_* + *c_all_* × *∆R*) – Annual deliveries × *B_all_*

(3) Estimated additional number of term births among ACS recipients

= Annual deliveries × (*B_all_* + *c_all_* × *∆R*) × (*B_term_* + *c_term_* × *∆R*) – Annual deliveries × *B_all_* × *B_term_*

Note: *∆R*, incremental change in the ACS/34w rate in the simulation scenario; *B_opt_*, baseline value of optimal-ACS/34w rate; *c_opt_*, regression coefficient of the ACS/34w rate in the multivariable model with an outcome of optimal-ACS/34w rate; *B_all_*, baseline value of ACS administration rate among all deliveries; *c_all_*, regression coefficient of the ACS/34w rate in the multivariable model with an outcome of ACS administration rate among all deliveries; *B_term_*, baseline value of term birth rate among ACS recipients; *c_term_*, regression coefficient of the ACS/34w rate in the multivariable model with an outcome of term/ACS proportion.

Second, we conducted a nationwide simulation in which all facilities included in the study with an ACS/34w rate <80% were hypothetically set to an 80% administration rate. Using the same model coefficients, we estimated aggregate changes in the number of cases for these three outcomes across the facilities included in this study. Furthermore, using anticipated absolute difference reported in a Cochrane review,^1^ we estimated that the increased ACS use under this scenario could prevent perinatal death in 2.3%, intraventricular hemorrhage (IVH) in 1.4%, and neurodevelopmental delay in childhood in 3.8% of the additional ACS recipients. Additionally, based on the absolute risk increase of 2.58% reported by Räikkönen et al.,^2^ we estimated the number of additional cases of any mental and behavioral disorders associated with the increase in term births following ACS exposure.

(1) Estimated additional number of prevented perinatal death

= Estimated additional number of ACS administration among all deliveries × 2.3%

(2) Estimated additional number of prevented IVH

= Estimated additional number of ACS administration among all deliveries × 1.4%

(3) Estimated additional number of prevented neurodevelopmental delay in childhood

= Estimated additional number of ACS administration among all deliveries × 3.8%

(4) Estimated additional number of any mental and behavioral disorders

= Estimated additional number of term births among ACS recipients × 2.58%

[1] McGoldrick E, Stewart F, Parker R, Dalziel SR. Antenatal corticosteroids for accelerating fetal lung maturation for women at risk of preterm birth. *Cochrane Database Syst Rev*. 2020; **12**(2): CD004454.

[2] Raikkonen K, Gissler M, Kajantie E. Associations Between Maternal Antenatal Corticosteroid Treatment and Mental and Behavioral Disorders in Children. *JAMA*. 2020; **323**(19): 1924-33.
